# SMRT regulation of nuclear receptors orchestrates bile acid homeostasis

**DOI:** 10.1038/s12276-026-01823-y

**Published:** 2026-09-09

**Authors:** Kyeongkyu Kim, Sungsoon Fang, Suk-Hyun Hong, Han Cho, Mathias Leblanc, Sandra Jacinto, Bernd Schnabl, Annette R. Atkins, Ruth T. Yu, Christopher Liddle, Morgan L. Truitt, Weiwei Fan, Chae Min Lee, Michael Downes, Ronald M. Evans

**Affiliations:** 1 Gene Expression Laboratory, Salk Institute for Biological Studies, La Jolla, CA USA; 2 Department of Biomedical Sciences and Graduate School of Medical Science, Brain Korea 21 Project, Gangnam Severance Hospital, Yonsei University College of Medicine, Seoul, Republic of Korea; 3 Department of Medicine, University of California San Diego, San Diego, CA USA; 4 Storr Liver Unit, Westmead Millennium Institute, University of Sydney, Sydney, Australia

**Keywords:** Metabolic diseases, Transcription

## Abstract

Nuclear receptors are key effectors of metabolic programs; however, the contribution of co-regulatory complexes in maintaining metabolic homeostasis is not fully understood. Here, we show that modulation of the constitutive androstane receptor (CAR) by the co-repressor silencing mediator of retinoic acid and thyroid hormone receptors (SMRT) is required for bile acid (BA) homeostasis. Transcriptional changes in the livers of mice harboring a targeted disruption in one of the nuclear receptor-interacting domains (RIDs), SMRT^mRID1^ mice, revealed increased expression of CAR target genes involved in BA export. Consistent with this, SMRT^mRID1^ mice challenged with a high-fat diet showed increased BAs in serum and urine with commensurate decreases in the liver and intestines. Notably, the reduction in intestinal BAs led to decreased activity of the BA receptor farnesoid X receptor (FXR). This reduction in FXR activity compromised the integrity of the intestinal barrier, increased bacterial burden, and augmented intestinal inflammation. Moreover, SMRT^mRID1^ mice were susceptible to intestinal damage, with higher mortality rates in acute colitis and colitis-associated cancer models. Treatment with a synthetic FXR agonist (XL335) rescued SMRT^mRID1^ mice during acute colitis. Collectively, these studies highlight the importance of SMRT in maintaining BA homeostasis through direct and indirect regulation of hepatic CAR and intestinal FXR activity, respectively, and point to potential therapeutic routes for combating intestinal pathologies associated with a high-fat diet.

## SMRT regulates bile acids and intestinal health in mice

Nuclear receptors (NRs) have a crucial role in gene transcription regulation, impacting metabolic homeostasis. This study explores the disruption of the silencing mediator for retinoid and thyroid hormone receptors (SMRT) network in high-fat diet (HFD)-fed SMRT^mRID1^ mice, revealing its impact on bile acid (BA) homeostasis and intestinal health. Researchers used SMRTmRID1 knock-in mice, which have mutations in the RID1 domain, to study the effects of impaired SMRT–NR interactions. They found that disrupted SMRT function derepresses hepatic constitutive androstane receptor activity, leading to altered BA metabolism and reduced intestinal BA levels. This reduction impairs farnesoid X receptor-mediated mucosal defense, increasing intestinal permeability and inflammation. The study highlights the importance of the SMRT–NR network in maintaining BA homeostasis and suggests that farnesoid X receptor activation could mitigate intestinal dysfunction. Future research could explore tissue-specific SMRT models to further understand these interactions. This summary was initially drafted using artificial intelligence, then revised and fact-checked by the author.

## Introduction

Nuclear receptors (NRs) collaborate with numerous transcriptional co-activators and co-repressors to precisely modulate the transcription of their target genes and maintain metabolic homeostasis in response to environmental and nutritional stresses^1^. Among these co-regulators, silencing mediator for retinoid and thyroid hormone receptors (SMRT) was initially identified as a transcriptional co-repressor that represses target gene expression mediated by unliganded NRs^2–5^. SMRT functions as a scaffold protein that recruits histone deacetylase-containing repression complexes, thereby establishing a repressive chromatin environment and silencing NR-dependent transcription^5^.

SMRT contains two distinct NR-interacting domains (RID1 and RID2), which mediate direct physical interactions with a broad range of NRs, including PPARs, TRs, RARs, farnesoid X receptor (FXR), and constitutive androstane receptor (CAR), enabling SMRT to exert context-dependent transcriptional repression across diverse metabolic pathways^6–8^. Through these interactions, SMRT critically regulates lipid and glucose metabolism, mitochondrial function, energy expenditure, and inflammatory responses. We previously reported that SMRT^mRID1^ mice, which harbor point mutations in the RID1 domain that selectively disrupt interactions with a subset of NRs, developed severe metabolic abnormalities, including extreme obesity and insulin resistance^9^. These findings demonstrated that the SMRT–NR transcriptional network has a crucial role in controlling the progression and severity of metabolic diseases^9^.

Although our previous work clearly established that defective SMRT–NR interactions drive profound metabolic dysfunction under high-fat diet (HFD) conditions, the broader physiological consequences of this disrupted co-repressor network remain incompletely understood. In particular, it remains unclear whether impairment of the SMRT–NR gene regulatory axis contributes to aberrant intestinal and enterohepatic homeostasis in HFD-fed SMRT^mRID1^ mice.

NRs such as FXR and CAR have central roles in the regulation of bile acid (BA) synthesis, transport, and detoxification, thereby maintaining enterohepatic BA homeostasis^10–12^. FXR has a well-established role in coordinating intestinal BA signaling and mucosal defense, whereas CAR regulates hepatic xenobiotic metabolism and influences BA transport and detoxification^10–12^. Because SMRT interacts with both FXR and CAR through its RID domains to repress their transcriptional activity, disruption of SMRT-mediated repression may profoundly alter BA metabolism and gut–liver crosstalk. However, whether dysregulation of the SMRT–CAR/FXR axis contributes to altered BA homeostasis and intestinal dysfunction associated with the metabolic phenotypes of SMRT^mRID1^ mice has not yet been explored.

Here, we associate disruption of the SMRT co-repressor network in HFD-fed SMRT^mRID1^ mice with derepression of hepatic CAR activity and subsequent disruption of enterohepatic BA homeostasis. Reduced intestinal BAs impaired FXR-dependent mucosal defense to promote intestinal inflammation and susceptibility to colitis-associated tumorigenesis.

## Materials and methods

### Animal studies

Generation of SMRT^mRID1^ knock-in mice was previously reported. Mice were maintained in a pathogen-free animal facility under a standard 12:12-h light/dark cycle. For diet-induced obesity, 8-week-old wild-type (WT) and SMRT^mRID1^ mice were fed either normal rodent chow (Labdiet, Catalog no. 5001, 4.5% fat, 4.00 kcal/g) or a HFD (Bio-Serv, Catalog no. F3282, 35.5% fat, 5.45 kcal/g) for 14 weeks. Body weight was recorded every week. For histology, 5 μm sections were stained with hematoxylin and eosin. For treatment with the synthetic FXR agonist XL335 (Cayman Chemical, Catalog no. 23350), XL335 was administered orally at a dose of 30 mg/kg daily. *Lactobacillus reuteri* was administered daily at a dose of 1 × 10^8^ PFU via oral gavage. Statistics were performed by Student’s *t* test. Values were presented as means and standard deviations. All protocols for mouse experiments were approved by the Institutional Animal Care and Use Committee of The Salk Institute.

### Murine colitis and colitis-associated tumorigenesis models

Dextran sulfate sodium (DSS) (MP Biomedicals; relative molecular mass 36,000–50,000, Catalog no. 160110) was provided in the drinking water at 5% (w/v) for 5 days, followed by 3 days of recovery with regular drinking water. In a separate experiment, after 3 weeks of treatment with either vehicle or XL335, mice were exposed to 2.5% DSS in the drinking water for 4 days, followed by a 3-day recovery period.

For colitis-associated tumorigenesis experiments, WT and SMRT^mRID1^ mice received intraperitoneal azoxymethane (AOM; 10 mg/kg) (Sigma, Catalog no. A5486) before initiation of HFD feeding and again after 8 weeks of HFD feeding, immediately before 2% DSS administration in the drinking water for 3 days. Mice were then maintained on HFD for an additional 8 weeks during the recovery period.

### Gene expression analysis

Total RNA was isolated from mouse tissues using TRIzol reagent (Invitrogen, Catalog no. 15596026) as per the manufacturer’s instructions. mRNA levels were quantified by qPCR with SYBR Green (Invitrogen, Catalog no. A25741). cDNA was synthesized from 1 μg of DNase-treated total RNA using SuperScript II reverse transcriptase (Invitrogen, Catalog no. 18064014). Samples were run in technical triplicates and relative mRNA levels were calculated by using the standard curve methodology and normalized against 36B4 mRNA levels in the same samples.

### 16S rDNA analysis

Bacterial genomic DNA was isolated using the QIAamp DNA Mini Kit (Qiagen, Catalog no. 51304), according to the manufacturer’s instructions. Universal 16S primers were used to quantify bacterial DNA abundance, and Rpl32 ribosomal protein (L32) primers were used to determine levels of mouse genomic DNA. The following mouse primer was used:

16S rDNA Forward 5ʹ-GTGGTGCATGGTTGTCGTCA-3ʹ,

16S rDNA Reverse 5ʹ-ACGTCGTCCCCACCTTCCTC-3ʹ.

### Histological analysis

Histology was scored by a pathologist in a blinded fashion as a combination of inflammatory cell infiltration (score 0–3) and tissue damage (score 0–3). The presence of occasional inflammatory cells in the lamina propria was scored as 0, increased numbers of inflammatory cells in the lamina propria were assigned score 1, confluence of inflammatory cells extending into the submucosa was scored as 2, and transmural extension of the infiltrate was scored as 3. For tissue damage, no mucosal damage was scored as 0, lymphoepithelial lesions were scored as 1, surface mucosal erosion or focal ulceration was scored as 2, and extensive mucosal damage and extension into deeper structures of the bowel wall was scored as 3. The combined histological score ranged from 0 (no changes) to 6 (extensive infiltration and tissue damage).

### Microarray analysis

Total RNA was isolated from liver tissue of WT and SMRT^mRID1^ mice. For each sample, 1 µg of total RNA (5 µl at 200 ng/µl) was labeled and hybridized to Affymetrix MG430 2.0 arrays according to the manufacturer’s standard protocols. Labeled samples were hybridized to microarray chips, scanned according to standard procedures, and gene expression data were processed using standard normalization and differential expression analysis pipelines.

### Chromatin immunoprecipitation (ChIP) and ChIP sequencing

Chromatin immunoprecipitation followed by sequencing (ChIP-seq) was performed using liver tissue of WT and SMRT^mRID1^ mice, as described previously^13^. Liver samples were crosslinked with 1% formaldehyde, quenched with glycine, and lysed. Chromatin was sheared by sonication to an average fragment size of 200–500 bp and immunoprecipitated using an anti-SMRT antibody (Abcam, ab5800) or control IgG. Following reverse crosslinking, ChIP DNA was purified and used for library preparation according to standard protocols. Libraries were sequenced on an Illumina platform, and reads were aligned to the reference genome.

### Serum FITC measurement

Serum fluorescein isothiocyanate (FITC) levels were measured to assess intestinal permeability. Mice were fasted for 6 h and were administered FITC–dextran orally (4 kDa, Sigma-Aldrich, Catalog no. 4694, 600 mg/kg). Blood was collected 4 h after gavage, and serum fluorescence was measured using a microplate reader (excitation: 485 nm; emission: 528 nm). FITC concentrations were calculated using a standard curve.

### Enzyme-linked immunosorbent assay

Serum amyloid A (SAA) levels were quantified using a commercially available mouse SAA enzyme-linked immunosorbent assay kit (Invitrogen, Catalog no. KMA0021), according to the manufacturer’s instructions. Absorbance was measured at 450 nm, and SAA concentrations were determined from a standard curve.

### Statistical analysis

For animal studies, sample size for experiments was determined empirically based on previous studies to ensure appropriate statistical power. Animals for each group of experiments were chosen randomly. No animals were excluded from statistical analysis, and the investigators were not blinded in the studies, except for histological scoring, which was performed in a blinded manner by a pathologist. Two-group comparisons were analyzed using unpaired two-tailed Student’s *t* tests. Multiple-group comparisons were analyzed using one-way or two-way analysis of variance with appropriate post hoc tests, as indicated in the figure legends. Survival analyses were performed using the log-rank test. Bars in graphs indicate mean ± STD. GraphPad Prism 9 was used for all analyses.

## Results

### Disruption of the SMRT RID1 domain derepresses hepatic CAR activity

**Fig. 1:**
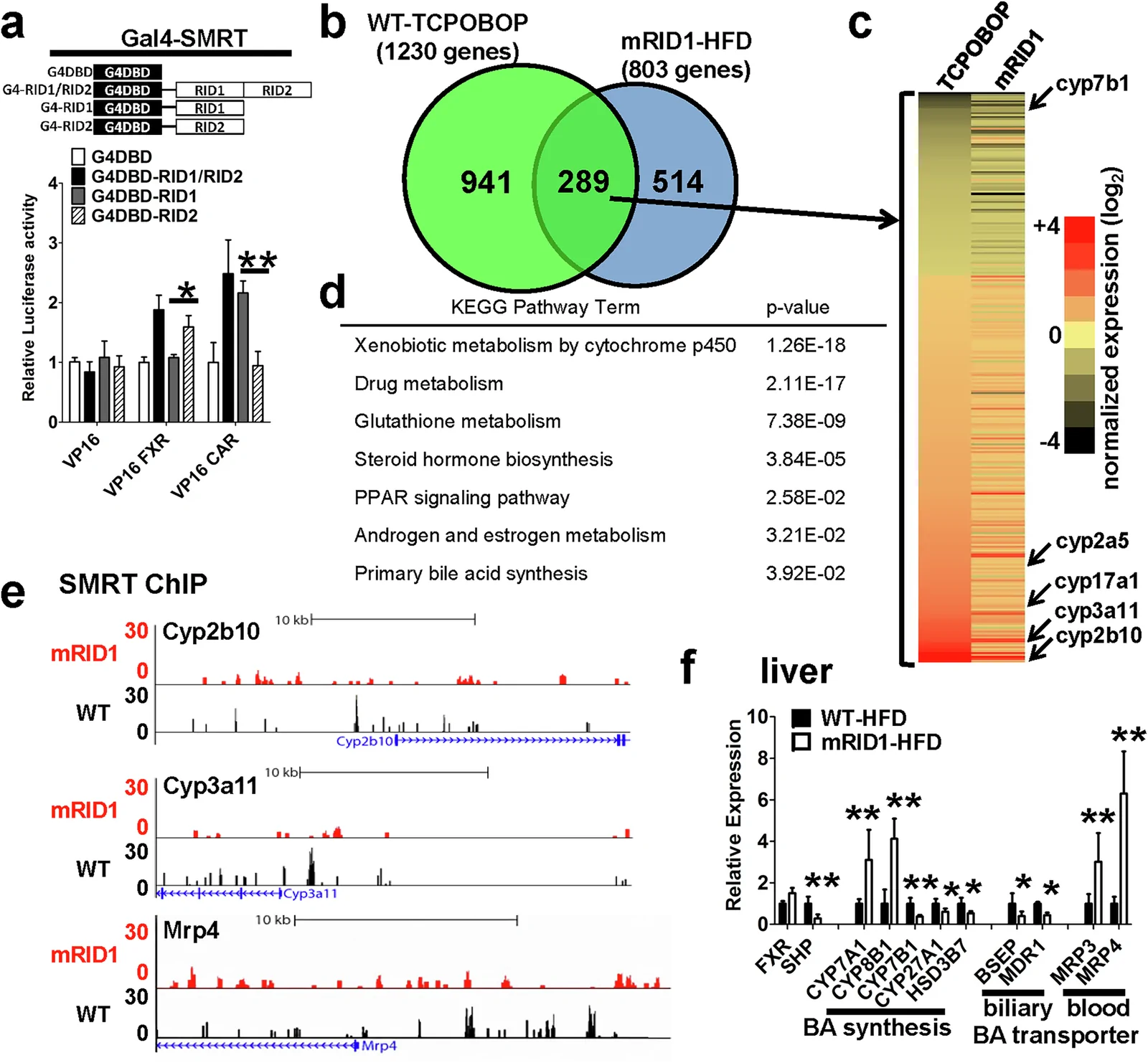
CAR activity is derepressed in the liver of HFD-fed SMRT^mRID1^ mice. **a** The two-hybrid assay showed that the transcriptional activity of constitutive androstane receptor (CAR) and farnesoid X receptor (FXR) are regulated by the RID domain of silencing mediator of retinoic acid and thyroid hormone receptor (SMRT). **b** Venn diagram showing the overlap between hepatic differentially expressed genes in high-fat diet (HFD)-fed SMRT^mRID1^ mice and HFD-fed wild-type (WT) controls and in TCPOBOP-treated WT mice and vehicle-treated WT controls. **c** Heatmap of the overlapping genes identified in part **b**; color denotes hepatic gene expression changes of HFD-fed SMRT^mRID1^ and TCPOBOP-treated mice. Selected genes are indicated on the right. **d** KEGG analysis for commonly changed hepatic genes in HFD-fed SMRT^mRID1^ and TCPOBOP-treated mice. **e** SMRT-bound peaks at the promoters of canonical CAR target genes and MRP4 gene are markedly altered in the livers of SMRT^mRID1^ mice compared with WT mice. The left axis indicates sequence-tag counts. **f** Hepatic gene expressions involved in bile acid (BA) homeostasis in HFD-fed WT and HFD-fed SMRT^mRID1^ mice. Data represent the mean ± standard deviation. Statistical analysis was performed with the Student’s *t* test. *P < 0.05, **P < 0.01. ChIP, chromatin immunoprecipitation.

Intriguingly, we observed that HFD-fed SMRT^mRID1^ mice showed shorter sleeping time than HFD-fed WT mice in phenobarbital-induced sleeping time assay (Supplementary Fig. 1), indicating that the rate of xenobiotic metabolism was higher in SMRT^mRID1^ mice. Phenobarbital is well established to trigger CAR-dependent transcriptional programs that promote hepatic xenobiotic metabolism^14,15^, and CAR can be repressed through interactions with the SMRT co-repressor^16^. Accordingly, we reasoned that impaired SMRT function in SMRT^mRID1^ mice may derepress CAR and potentially augment CAR transcriptional activity. To determine whether SMRT directly interacts with CAR to regulate its transcriptional activity, a mammalian two-hybrid assay was performed using expression vectors of VP16-conjugated NRs and different RID fragments of SMRT. Although FXR interacted with RID2, CAR directly interacted with RID1 (Fig. 1a). These in vitro data demonstrate that the RID1 domain of SMRT is critical for controlling CAR transcriptional activity. We therefore hypothesized that SMRT directly interacts with CAR through the RID1 domain to repress its transcriptional activity and that disruption of this interaction derepresses CAR, leading to enhanced expression of CAR target genes in SMRT^mRID1^ mice.

To determine whether derepressed CAR activity in SMRT^mRID1^ mice resembles ligand-induced CAR activation, we examined microarray analyses of in vivo hepatic gene expression changes in HFD-fed SMRT^mRID1^ mice (normalized to HFD-fed WT controls) and in WT mice treated with the CAR agonist TCPOBOP (normalized to vehicle-treated WT controls). Consistently, gene expression changes in SMRT^mRID1^ mice overlapped with those of TCPOBOP-treated WT mice (289 out of 803 genes, 35.9%) (Fig. 1b). Notably, the overlapping gene expression patterns also showed remarkable similarity (Fig. 1c). As expected, CAR-mediated drug metabolism was the most prominently shared pathway between SMRT^mRID1^ and TCPOBOP-treated WT mice (Fig. 1d), suggesting that SMRT RID1 domain has a key role in SMRT interaction with CAR to repress its transcriptional activity.

We next examined whether SMRT interacts with CAR at the promoters of CAR target genes. Robust SMRT occupancy was observed at the promoters of canonical CAR target genes, including *Cyp2b10* and *Cyp3a11*, in WT mice, whereas SMRT binding at these loci was markedly diminished in SMRT^mRID1^ mice (Fig. 1e). These findings are consistent with the in vitro two-hybrid assays and the in vivo global hepatic gene expression analyses.

It is widely accepted that CAR has a key role in coordinating BA homeostasis as well as drug metabolism in concert with other NRs, including FXR. Recent studies have shown that BA homeostasis serves as a general metabolic integrator regulating energy expenditure and protecting against obesity and insulin resistance. Because HFD-fed SMRT^mRID1^ mice develop severe obesity and metabolic defects, including insulin resistance, we next examined hepatic gene expression profiles involved in BA homeostasis.

Our microarray analysis revealed that the primary BA synthesis pathway is significantly altered in HFD-fed SMRT^mRID1^ mice (*P* = 0.0392; Fig. 1d). We found that expression of small heterodimer partner (*SHP*), a key orphan NR regulating BA synthesis, was markedly reduced in SMRT^mRID1^ mice (Fig. 1f). Although the expression of BA synthesis genes, including *Cyp7b1* and *Cyp27a1*, was suppressed, the SHP-repressed genes *Cyp7a1* and *Cyp8b1* were robustly upregulated in SMRT^mRID1^ mice (Fig. 1f). Strikingly, the CAR-induced BA efflux transporter genes *Mrp3* and *Mrp4*, which mediate BA export from the liver into the circulation, were markedly increased in SMRT^mRID1^ mice (Fig. 1f).

Consistent with other CAR target genes, robust SMRT occupancy was detected at the *Mrp4* promoter in WT mice, whereas SMRT binding was markedly diminished in SMRT^mRID1^ mice (Fig. 1e). These findings indicate that BA efflux transporters are directly regulated by CAR and that CAR activity is derepressed in SMRT^mRID1^ mice.

Taken together, these data strongly support a critical role for the SMRT RID1 domain in mediating SMRT–CAR interaction and repressing CAR transcriptional activity.

### Reduced intestinal BA levels in SMRT^mRID1^ mice impair mucosal defense and barrier integrity

**Fig. 2:**
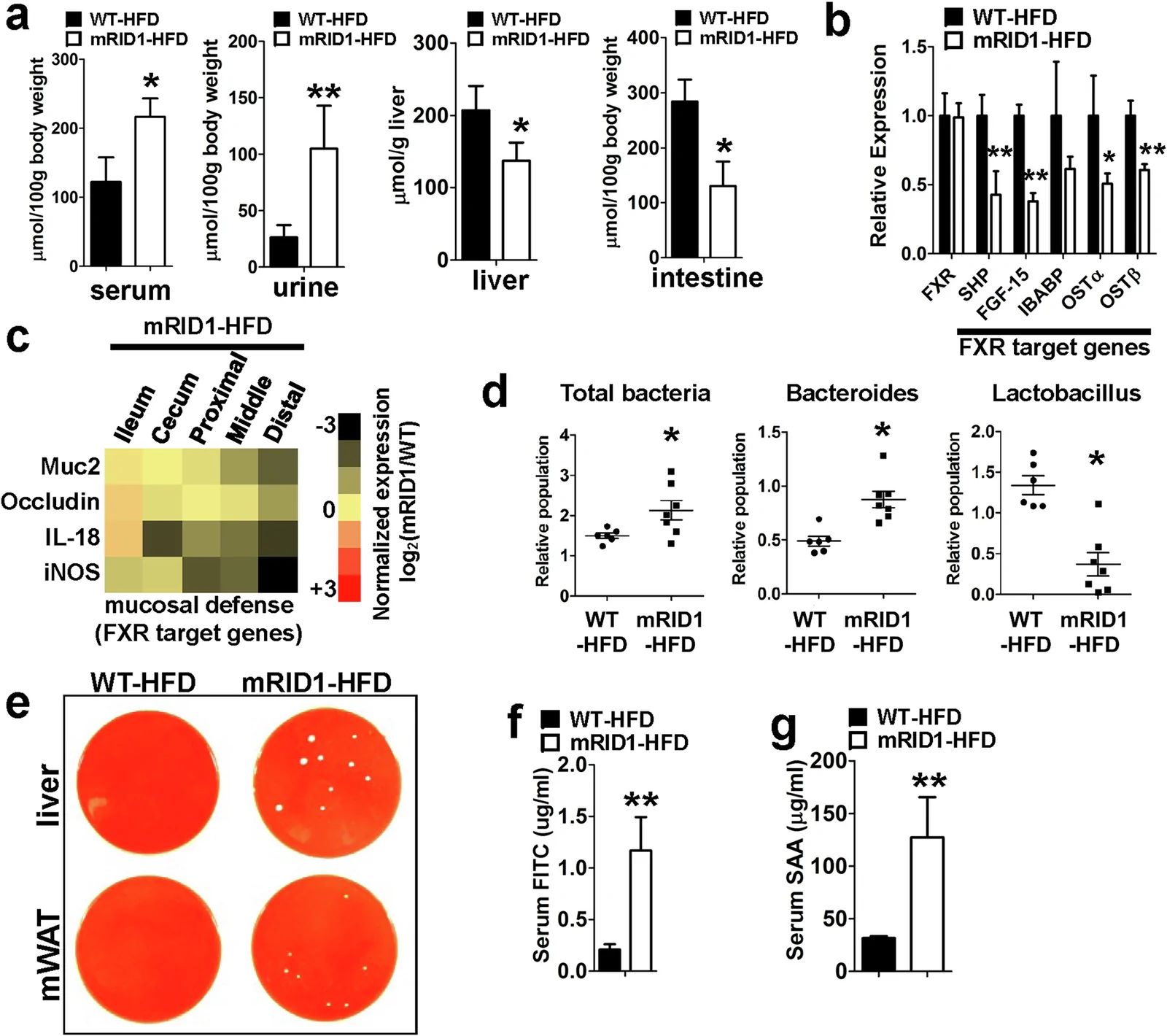
Transcriptional repression is required for BA homeostasis and microbial ecology to maintain intestinal homeostasis. **a** Bile acid (BA) levels in the serum, urine, liver, and intestine. **b** Hepatic gene expressions involved in BA homeostasis in HFD-WT and HFD-fed SMRT^mRID1^ mice. **c** Farnesoid X receptor (FXR)-mediated mucosal defense gene expressions in HFD-fed SMRT^mRID1^ mice relative to WT mice. **d** Fecal bacterial analysis by qPCR in HFD-fed WT and SMRT^mRID1^ mice. **e** Gut bacterial translocation from gut to liver or mesenteric white adipose tissue (mWAT) in HFD-fed SMRT^mRID1^ mice. **f** Intestinal permeability. **g** Serum amyloid A for bacterial infection indicator. Data represent the mean ± standard deviation. Statistical analysis was performed with the Student’s *t* test. *P < 0.05, **P < 0.01. FITC, fluorescein isothiocyanate; SHP, small heterodimer partner; SMRT, silencing mediator of retinoic acid and thyroid hormone receptor.

To determine whether these transcriptional changes translate into altered BA homeostasis in vivo, we next measured BA levels in multiple tissues. Consistent with the gene expression profiles, serum BA levels were markedly elevated in SMRT^mRID1^ mice, accompanied by increased urinary BA excretion, whereas hepatic and intestinal BA levels were significantly reduced (Fig. 2a). These in vivo BA measurements indicate that disruption of the SMRT-NR hepatic gene network leads to aberrant enterohepatic BA homeostasis, characterized by enhanced urinary BA loss and a reduced intestinal BA pool. Such alterations are likely to contribute to the metabolic defects previously observed in HFD-fed SMRT^mRID1^ mice, including obesity, reduced energy expenditure, and insulin resistance^9^.

BAs serve as ligands for the BA receptor FXR and thereby regulate the expression of mucosal defense genes required to maintain intestinal homeostasis. Previous studies demonstrated that obstruction of BA flow results in bacterial proliferation and mucosal injury leading to the translocation of bacteria across the epithelial barrier and systemic infection^17–19^. These pathologies were caused by impaired FXR transcriptional activity, which resulted from depletion of ligand availability in intestine, leading to reduced gene expressions for mucosal defense. Thus, we hypothesized that reduced intestinal BA levels in HFD-fed SMRT^mRID1^ mice would impair FXR transcriptional activity and consequently disrupt the expression of FXR target genes involved in mucosal defense.

We found that the expression of well-established FXR target genes was markedly reduced in the intestine of SMRT^mRID1^ mice (Fig. 2b). Consistent with previous studies, reduced intestinal BA levels were associated with decreased expression of FXR-mediated mucosal defense genes, suggesting that SMRT^mRID1^ mice may exhibit an altered microbial ecology (Fig. 2c). We therefore investigated whether the profound reduction in mucosal defense gene expression resulted in dysbiosis in SMRT^mRID1^ mice.

Fecal samples were collected from single-housed, HFD-fed WT, and SMRT^mRID1^ mice to analyze bacterial DNA content. Significant alterations in the gut microbiota were observed in SMRT^mRID1^ mice, with increased bacterial abundance detected in fecal samples (Fig. 2d). Higher levels of bacteria were also detected in peripheral tissues, including the liver and mesenteric adipose tissue, suggesting increased intestinal permeability in SMRT^mRID1^ mice (Fig. 2e).

To directly assess mucosal barrier integrity, intestinal permeability was quantitatively measured by oral administration of FITC-dextran followed by serum fluorescence quantification 4 h after gavage. Serum FITC fluorescence was substantially elevated in SMRT^mRID1^ mice compared with WT controls (Fig. 2f).

To obtain further evidence of bacterial translocation, we measured serum levels of SAA, a marker of acute bacterial infection, as well as multiple cytokines and chemokines. Consistent with enhanced bacterial translocation, serum concentrations of SAA and the chemokines, G-CSF, KC, and MCP-1 were increased in HFD-fed SMRT^mRID1^ mice (Fig. 2g and Supplementary Fig. 2). In addition, both serum concentrations and gene expression levels of the pro-inflammatory cytokines IL-6 and tumor necrosis factor-α were elevated in HFD-fed SMRT^mRID1^ mice (Supplementary Figs. 2 and 3).

Collectively, these findings support a model in which reduced intestinal BA levels impair FXR-mediated mucosal defense gene regulation, leading to dysbiosis, increased intestinal permeability, and intestinal inflammation in HFD-fed SMRT^mRID1^ mice.

### SMRT^mRID1^ mice are sensitized to intestinal damage

**Fig. 3:**
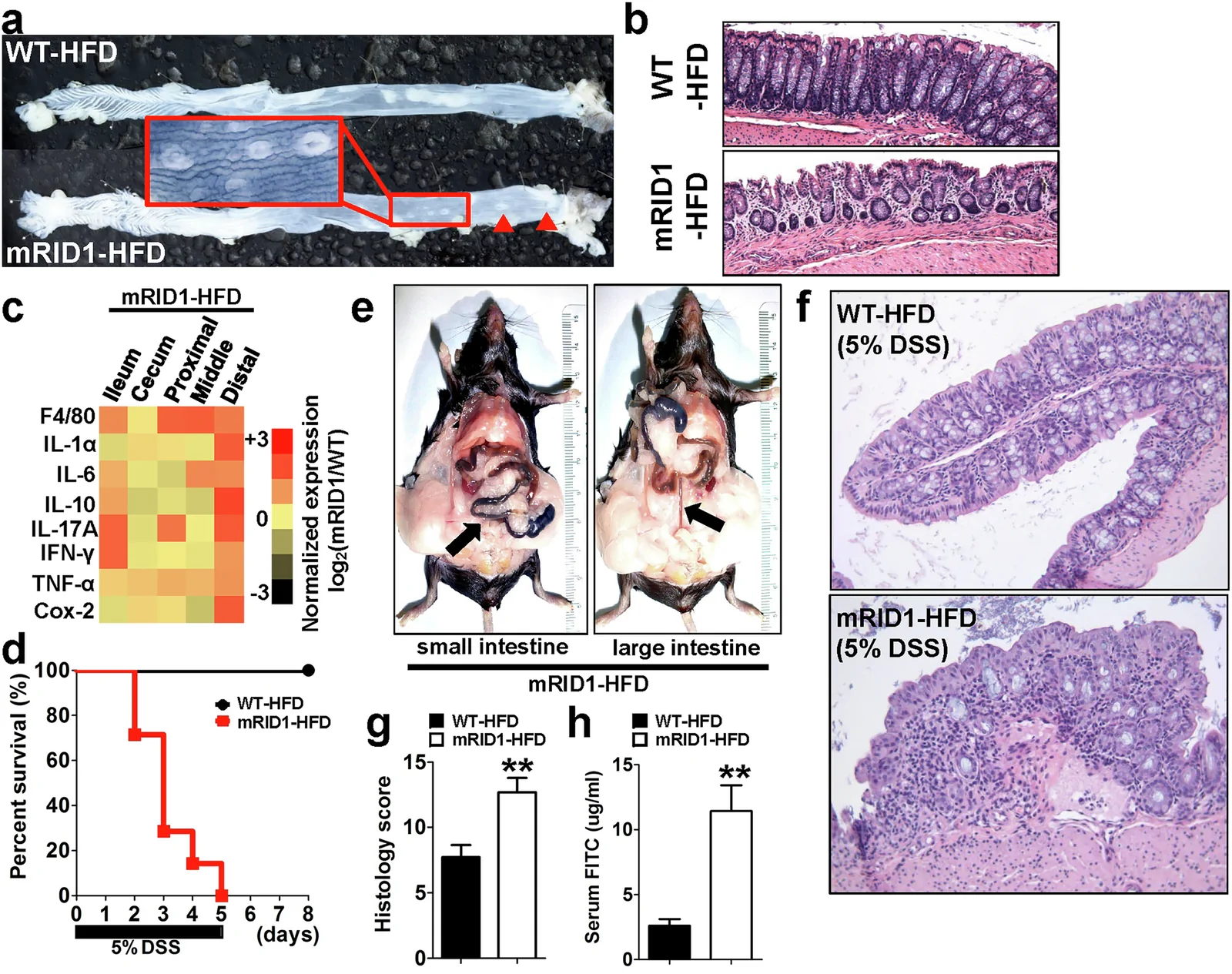
SMRT^mRID1^ mice develop severe intestinal pathologies with high-fat diet. **a** Colonic ulcers in high-fat diet (HFD)-fed SMRT^mRID1^ mice. **b** Hematoxylin and eosin staining of histological sections of colons. **c** Inflammatory cytokine expressions of gastrointestinal tract (ileum to distal colon) in HFD-fed wild-type (WT) and SMRT^mRID1^ mice. **d**–**h** To induce acute colitis, HFD-fed WT and SMRT^mRID1^ mice were given 5% dextran sulfate sodium (DSS) in the drinking water for 5 days. **d** Survival rate. **e** Macroscopic view of the bleeding intestinal tract in DSS-challenged HFD-fed SMRT^mRID1^ mice. **f** Hematoxylin and eosin staining of histological sections from colons in DSS-challenged HFD-fed WT and SMRT^mRID1^ mice. **g** Histological score. **h** Intestinal permeability. Data represent the mean ± standard deviation. Statistical analysis was performed with Student’s *t* test (parts **g** and **h**). *P < 0.05 and **P < 0.01. FITC, fluorescein isothiocyanate; IFN, interferon; SMRT, silencing mediator of retinoic acid and thyroid hormone receptor; TNF, tumor necrosis factor.

We next examined whether HFD feeding induces chronic intestinal inflammation in SMRT^mRID1^ mice. Although SMRT^mRID1^ mice appeared grossly normal when maintained on normal chow (Supplementary Fig. 4), HFD-fed SMRT^mRID1^ mice occasionally developed severe diarrhea (Supplementary Fig. 5a). Strikingly, HFD-fed SMRT^mRID1^ mice exhibited colonic ulcers in the distal colon with 100% penetrance (Fig. 3a).

Histological analysis revealed a depletion of mucin-producing goblet cells and distortion of crypt architecture in the colonic epithelium surrounding ulcerated regions in SMRT^mRID1^ mice (Fig. 3b and Supplementary Fig. 5b). Consistent with the presence of colonic ulceration, expression of pro-inflammatory cytokines was significantly elevated throughout the gastrointestinal tract, with particularly pronounced increases in the distal colon of SMRT^mRID1^ mice (Fig. 3c). These findings indicate that HFD induces chronic intestinal inflammation, predominantly in the distal colon of SMRT^mRID1^ mice, a phenotype that resembles ulcerative colitis in human patients.

It is well recognized that physiological inflammation in the intestine is protective, whereas excessive production of inflammatory cytokines contributes to colitis pathogenesis. We therefore hypothesized that HFD-fed SMRT^mRID1^ mice would be hypersusceptible to intestinal injury in a dextran sodium sulfate (DSS)-induced acute colitis model. Whereas no mortality was observed in WT mice, HFD-fed SMRT^mRID1^ mice exhibited severe weight loss, increased mortality, gastrointestinal bleeding, and significant shortening of the colon during DSS-induced colitis (Fig. 3d, e and Supplementary Fig. 6). Histological examination of colon sections from SMRT^mRID1^ mice revealed pronounced crypt distortion, crypt inflammation, extensive immune cell infiltration into the lamina propria, and increased intestinal permeability (Fig. 3f–h). Collectively, these data demonstrate that SMRT^mRID1^ mice mount exaggerated local and systemic immune responses during DSS-induced acute colitis.

The intestinal microbiota is a fundamental determinant of mucosa-associated immune responses, and inappropriate host responses to commensal microorganisms can trigger chronic inflammatory disorders^17^. Previous studies have shown that supplementation with lactobacilli reduces inflammatory cytokine expression and attenuates the severity of colitis^18^. Because HFD-fed SMRT^mRID1^ mice showed a marked reduction in lactobacilli (Fig. 2d), we next tested whether *L. reuteri* supplementation could mitigate their heightened susceptibility to DSS-induced colitis. During acute colitis induced by 2.5% DSS, HFD-fed SMRT^mRID1^ mice exhibited poorer survival than HFD-fed WT mice, whereas *L. reuteri* supplementation partially improved survival in SMRT^mRID1^ mice (Supplementary Fig. 7a). In parallel, serum endotoxin levels were reduced in *L. reuteri*-supplemented SMRT^mRID1^ mice compared with unsupplemented SMRT^mRID1^ mice (Supplementary Fig. 7b). Histological examination of the distal colon further showed that live *L. reuteri*, but not heat-killed *L. reuteri*, attenuated epithelial damage in DSS-treated WT and SMRT^mRID1^ mice (Supplementary Fig. 7c). These findings suggest that the loss of lactobacilli contributes, at least in part, to the exacerbated intestinal injury and inflammation observed in HFD-fed SMRT^mRID1^ mice.

### Loss of SMRT co-repression promotes colitis-associated colorectal tumorigenesis

**Fig. 4:**
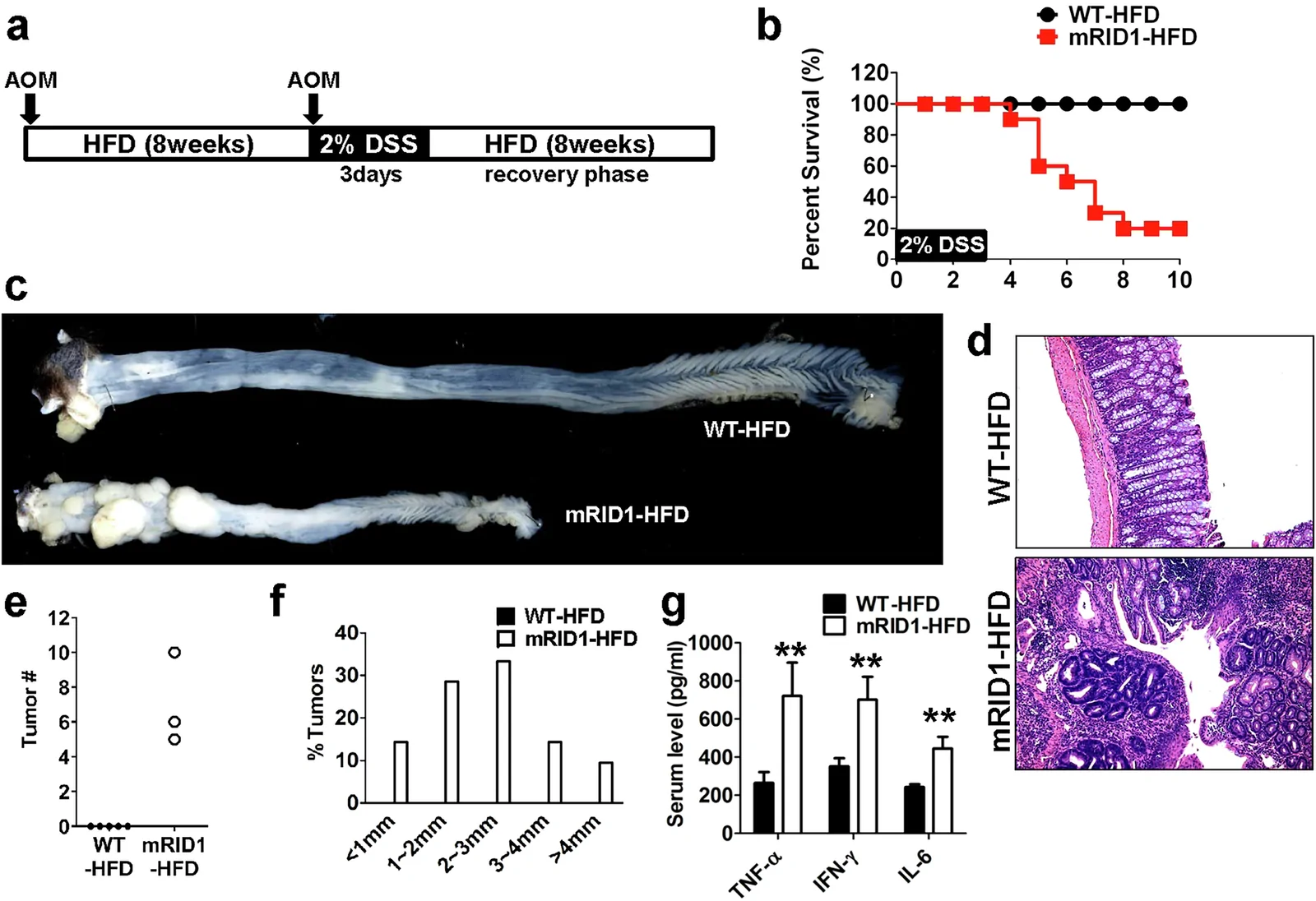
Tumorigenesis is exacerbated in SMRT^mRID1^ mice during colitis-associated colon cancer. **a** Scheme of colitis-associated colon cancer model. **b** Survival rate during dextran sulfate sodium (DSS) treatment. **c** Severe tumor burdens in high-fat diet (HFD)-fed SMRT^mRID1^ mice. **d** Hematoxylin and eosin staining of histological sections of tumors. No tumors were observed in wild-type (WT) mice. **e** Tumor numbers in HFD-fed WT and SMRT^mRID1^ mice. **f** Tumor size distribution. **g** Inflammatory cytokine levels in the serum. Data represent the mean ± standard deviation. Statistical analysis was performed with the Student’s *t* test. *P < 0.05 and **P < 0.01. AOM, azoxymethane; IFN, interferon; SMRT, silencing mediator of retinoic acid and thyroid hormone receptor; TNF, tumor necrosis factor.

Inflammatory bowel disease is an established risk factor for colorectal tumorigenesis^19,20^. We therefore sought to determine whether HFD-induced intestinal inflammation promotes colitis-associated colon cancer in SMRT^mRID1^ mice. HFD-fed WT and SMRT^mRID1^ mice were subjected to an AOM plus DSS regimen. Because SMRT^mRID1^ mice are hypersusceptible to DSS-induced intestinal inflammation, a single DSS cycle was administered instead of the conventional three repeated cycles (Fig. 4a).

Despite using this reduced DSS protocol, SMRT^mRID1^ mice exhibited substantial mortality during DSS treatment, necessitating further dose reduction (Fig. 4b). Whereas WT mice rarely developed tumors following a single low-dose DSS cycle, SMRT^mRID1^ mice displayed a higher tumor burden, including increased tumor number and size (Fig. 4c–f). Consistent with enhanced tumorigenesis, serum levels of inflammatory cytokines were markedly elevated in SMRT^mRID1^ mice (Fig. 4g).

Collectively, these results indicate that disruption of the SMRT–NR gene network impairs BA homeostasis and microbial ecology, thereby promoting intestinal inflammation and increasing susceptibility to colitis-associated colorectal cancer under environmental stress such as HFD.

### FXR activation attenuates intestinal dysfunction in SMRT^mRID1^ mice

**Fig. 5:**
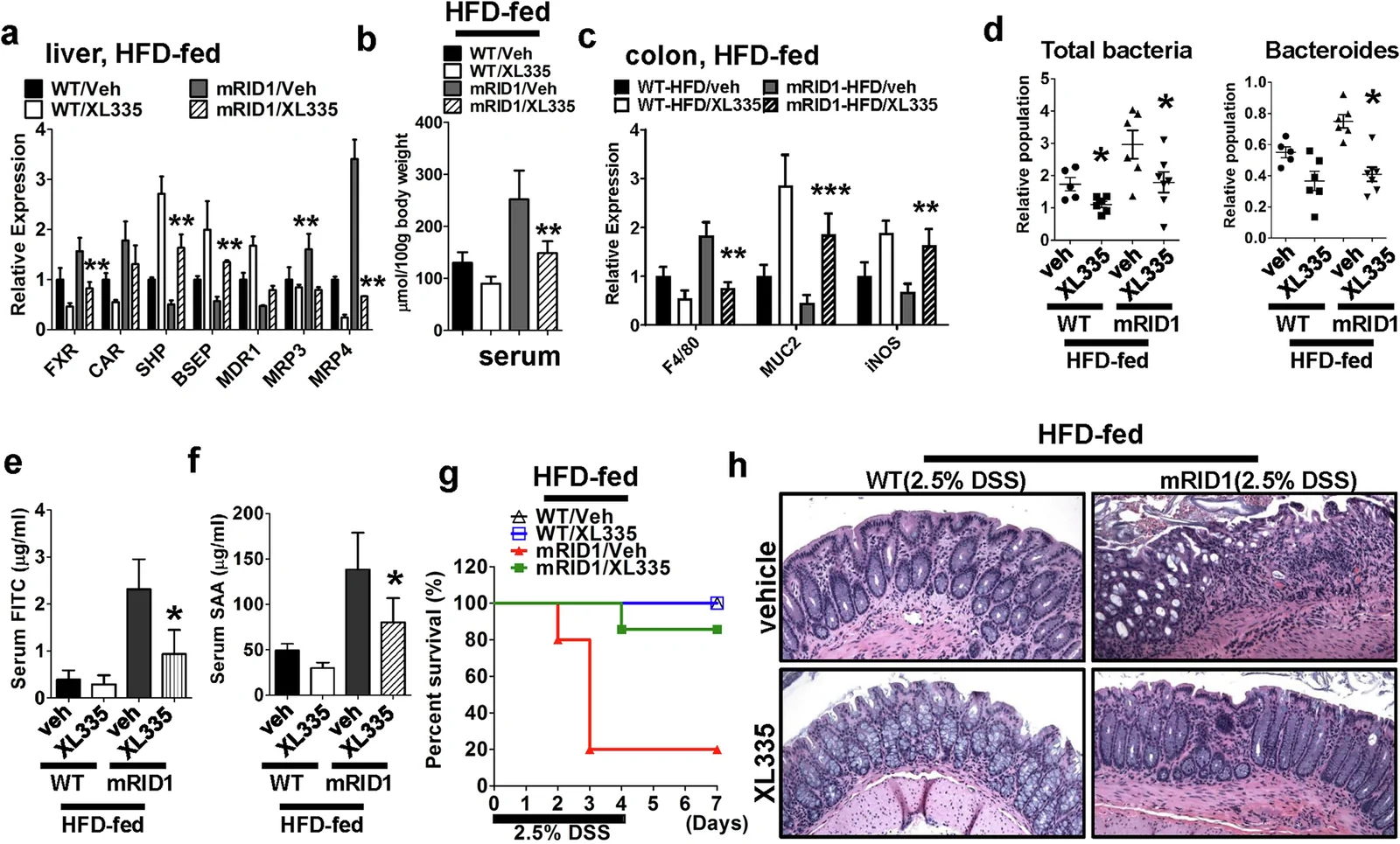
FXR activation rescues HFD-fed SMRT^mRID1^ mice from intestinal pathologies. **a**–**f** High-fat diet (HFD)-fed wild-type (WT) and SMRT^mRID1^ mice received either vehicle or farnesoid X receptor (FXR) agonist XL335 (30 mg/kg) daily for 3 weeks. **a** Hepatic gene expression of bile acid (BA) homeostasis. **b** BA levels in serum. **c** Colonic expression of FXR target genes. **d** Fecal bacterial analysis by qPCR. **e** Intestinal permeability. **f** Serum amyloid A for bacterial infection indicator. **g**, **h** To induce acute colitis, mice received 2.5% dextran sulfate sodium (DSS) in the drinking water for 4 days after 3-week treatment of either vehicle or XL335. **g** Survival rate. **h** Hematoxylin and eosin staining of histological sections from colons in 2.5% DSS-challenged WT and SMRT^mRID1^ mice. Data represent the mean ± standard deviation. Statistical significance was determined by two-way analysis of variance followed by Tukey’s post hoc test to compare the vehicle and XL335 groups. *P < 0.05, **P < 0.01, ***P < 0.001. FITC, fluorescein isothiocyanate; SMRT, silencing mediator of retinoic acid and thyroid hormone receptor.

Finally, we examined whether reactivation of FXR could restore mucosal defense gene expression and attenuate intestinal pathologies in SMRT^mRID1^ mice. To this end, HFD-fed mice were treated daily with vehicle or the synthetic FXR agonist XL335 (30 mg/kg) for 3 weeks. We first assessed whether FXR activation restored gene expression programs involved in enterohepatic BA circulation. Notably, expression of *Mrp4* was suppressed in XL335-treated mice, accompanied by reduced serum and urinary BA levels, indicating that FXR activation restores enterohepatic BA homeostasis (Fig. 5a, b). This aligns with prior work showing that ligand-mediated activation of FXR represses MRP4 by competing with CAR at an overlapping FXR/CAR response element in the MRP4 promoter^20^.

We next examined whether FXR activation induces mucosal defense gene expression. Activation of FXR robustly increased the expression of mucosal defense genes while concomitantly reducing macrophage marker genes in the ileum and colon (Fig. 5c and Supplementary Fig. 8), consistent with the established role of FXR as a key regulator of intestinal mucosal defense^17^. In agreement with these molecular changes, commensal bacterial overgrowth was reduced in XL335-treated mice compared with vehicle-treated controls (Fig. 5d). Furthermore, intestinal permeability and serum levels of SAA were decreased following XL335 treatment (Fig. 5e, f).

We then tested whether FXR activation could rescue HFD-fed SMRT^mRID1^ mice from DSS-induced acute colitis. Strikingly, XL335 treatment significantly reduced mortality in HFD-fed SMRT^mRID1^ mice during DSS challenge, accompanied by improved intestinal epithelial barrier integrity (Fig. 5g, h).

Collectively, these findings demonstrate that FXR reactivation restores mucosal defense gene expression, suppresses intestinal inflammation, and preserves intestinal barrier function, thereby maintaining intestinal homeostasis in SMRT^mRID1^ mice.

## Discussion

Transcriptional activities of NRs are tightly controlled by co-activators and co-repressors, and disruption of these regulatory interactions can destabilize tissue homeostasis under environmental stress^21–24^. In the intestine, maintenance of homeostasis depends on balanced interactions among epithelial cells, immune responses, and commensal microbiota^25–28^. Our findings identify the SMRT–NR co-repressor network as a critical regulator of this balance by preserving enterohepatic BA homeostasis and protecting against intestinal inflammation under HFD conditions.

The HFD dependence of the SMRT^mRID1^ intestinal phenotype likely reflects the increased physiological demand for efficient enterohepatic BA recycling during high-fat feeding^29,30^; under these conditions, derepressed CAR activity diverts BAs away from the intestine and unmasks barrier dysfunction and colitis. Paradoxically, SMRT^mRID1^ mice develop obesity despite intestinal BA depletion, consistent with our previous finding that HFD-induced metabolic abnormalities in these mice reflect broader disruption of SMRT–NR signaling across multiple metabolic tissues^9^. Thus, reduced intestinal FXR activity is likely a major driver of the intestinal inflammatory phenotype, whereas the obesity phenotype more likely reflects systemic metabolic reprogramming^11,31^. These represent two distinct consequences that arise from the same underlying defect in SMRT-dependent NR regulation.

Mechanistically, HFD led to dramatic induction of CAR-mediated BA efflux to the circulation, resulting in BA depletion in the intestine. BA homeostasis is unique to vertebrates as it provides the host with the ability to digest and utilize various dietary fats. BAs can also enhance intestinal antimicrobial defense by activating FXR^17–19^, the key regulator of mucosal defense gene expression. Thus, intestinal BA reduction led to decreased FXR transcriptional activities resulting in aberrant microbial ecology and intestinal inflammation. In accordance with our findings, another group also reported that obstruction of bile flow resulted in commensal overgrowth and mucosal injury in the intestine that led to the bacterial translocation across the epithelial barrier and systemic inflammation^17^. In that study, mice lacking FXR transcriptional activity had increased commensal bacterial growth and a compromised epithelial barrier, suggesting the central role of FXR to protect the intestine from bacterial invasion.

The *L. reuteri* rescue further supports the idea that a BA–microbiome axis operates downstream of SMRT dysfunction. Reduced intestinal FXR activity would be expected to weaken epithelial defense while creating a microbial environment less supportive of beneficial commensals^32^. The partial rescue by live, but not heat-killed, *L. reuteri* suggests that restoring host–microorganism crosstalk can ameliorate epithelial injury without directly correcting the upstream hepatic defect.

These findings also have translational relevance, as reduced intestinal FXR activity and BA dysregulation have been reported in patients with inflammatory bowel disease. More broadly, disruption of the BA–microbiome–gut–liver axis is increasingly implicated in human inflammatory and metabolic disorders.

Although the rescue by XL335 and *L. reuteri* strongly supports hepatic BA misdistribution as a major driver of the phenotype, the global nature of the SMRT^mRID1^ mutation means that a direct contribution of SMRT dysfunction within intestinal epithelial cells cannot be excluded. Future studies using tissue-specific SMRT models will be required to resolve this issue.

**Fig. 6:**
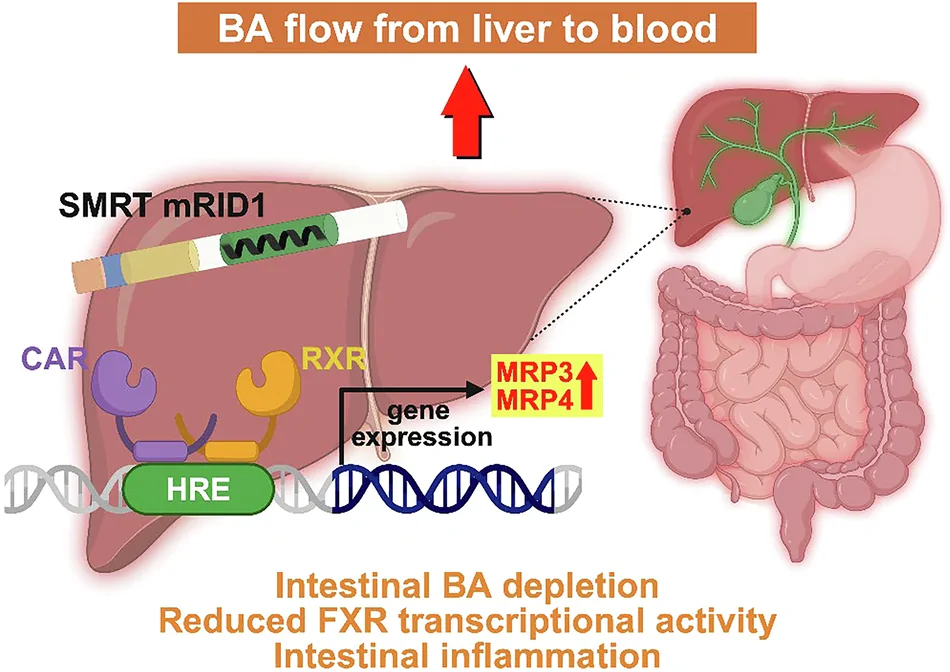
Proposed model linking SMRT dysfunction to BA imbalance and intestinal inflammation. Schematic model summarizing how silencing mediator of retinoic acid and thyroid hormone receptor (SMRT) dysfunction promotes hepatic bile acid (BA) efflux via constitutive androstane receptor (CAR)-dependent induction of MRP3 and MRP4, leading to intestinal BA depletion, reduced FXR signaling, and intestinal inflammation.

Altogether, our findings demonstrate that the SMRT–NR gene network strictly regulates the hepatic BA efflux transporters MRP3 and MRP4 to maintain enterohepatic BA homeostasis (Fig. 6). Disruption of SMRT–NR interactions in SMRT^mRID1^ mice, especially with environmental stress such as HFD, impeded enterohepatic BA recycling, resulting in intestinal BA depletion, leading to reduced intestinal FXR transcriptional activity, altered microbial ecology, and intestinal inflammation. Furthermore, administration of a synthetic FXR agonist in vivo ameliorated these phenotypes, underscoring the importance of transcriptional repression through the NR-interacting co-repressor SMRT in the maintenance of BA homeostasis. These findings emphasize the importance of NR crosstalk between CAR and FXR in the liver. Therapeutic strategies that account for CAR/FXR crosstalk in maintaining BA homeostasis and restoring microbial homeostasis may prove valuable for managing some of the clinical manifestations commonly associated with intestinal inflammation.

## Supplementary information

Supplementary Information
