# Supplementary material for: SMRT regulation of nuclear receptors orchestrates bile acid homeostasis
Source: Exp Mol Med. 2026 Sep 9;58(9):3088–96. doi: 10.1038/s12276-026-01823-y (PMC13633267; doi:10.1038/s12276-026-01823-y)
Supplement: Supplementary file 1 [file 12276_2026_1823_MOESM1_ESM.pdf]

SMRT Regulation of Nuclear Receptors Orchestrates Bile Acid Homeostasis

Kyeongkyu Kim[1,*], Sungsoon Fang[1,2,*], Suk-hyun Hong[1], Han Cho[1], Mathias Leblanc[1], Sandra Jacinto[1], Bernd Schnabl[3], Annette Atkins[1], Ruth T. Yu[1], Christopher Liddle[4], Morgan L. Truitt[1], Weiwei Fan[1], Chae Min Lee[2], Michael Downes[1,†] and Ronald M. Evans[1†]

[1]Gene Expression Laboratory, Salk Institute for Biological Studies, La Jolla, California, USA

[2]Department of Biomedical Sciences and Graduate School of Medical Science, Brain Korea 21 Project, Gangnam Severance Hospital, Yonsei University College of Medicine, Republic of Korea

[3]Department of Medicine, University of California San Diego, California, USA,

[4]Storr Liver Unit, Westmead Millennium Institute, University of Sydney, Australia

[*]These authors equally contributed to this work

[†] Corresponding author:

Michael Downes and Ronald M. Evans,

Gene Expression Laboratory, The Salk Institute for Biological Studies

10010 N. Torrey Pines Rd., La Jolla, CA 92037

E-mail: downes@salk.edu and evans@salk.edu

Supplementary Information

Contents:

Materials and Methods

Supplementary Figures

Supplementary Figure 1. HFD-fed SMRT^{mRID1} mice exhibit reduced sleep duration compared to WT mice

Supplementary Figure 2. Serum levels of inflammatory cytokines are elevated in HFD-fed SMRT^{mRID1} mice

Supplementary Figure 3. Altered intestinal gene expression in HFD-fed SMRT^{mRID1} mice

Supplementary Figure 4. Intestinal morphology and barrier function are preserved in SMRT^{mRID1} mice under normal chow conditions.

Supplementary Figure 5. Severe diarrhea and colonic epithelial damage in HFD-fed SMRT^{mRID1} mice

Supplementary Figure 6. HFD-fed SMRT^{mRID1} mice display increased susceptibility to DSS-induced acute colitis

Supplementary Figure 7. *Lactobacillus reuteri* supplementation partially ameliorates DSS-induced intestinal inflammation in HFD-fed SMRT^{mRID1} mice

Supplementary Figure 8. FXR activation restores entero-hepatic bile acid homeostasis and mucosal defense gene expression in HFD-fed SMRT^{mRID1} mice

Materials and Methods

Phenobarbital-induced sleep assay

Phenobarbital-induced sleep time was measured as previously described[1]. Mice were administered phenobarbital by intraperitoneal injection. The sleeping mice were placed on their backs, and sleep time was measured until the animals had regained enough consciousness to right itself repeatedly.

Goblet cell staining

Goblet cells in intestinal tissues were visualized by histological staining. Briefly, intestinal segments were fixed in 10% neutral-buffered formalin, embedded in paraffin, sectioned, and stained with Periodic acid–Schiff (PAS) to detect mucin-producing goblet cells according to standard protocols. Stained sections were examined under a light microscope, and representative images were acquired for analysis.

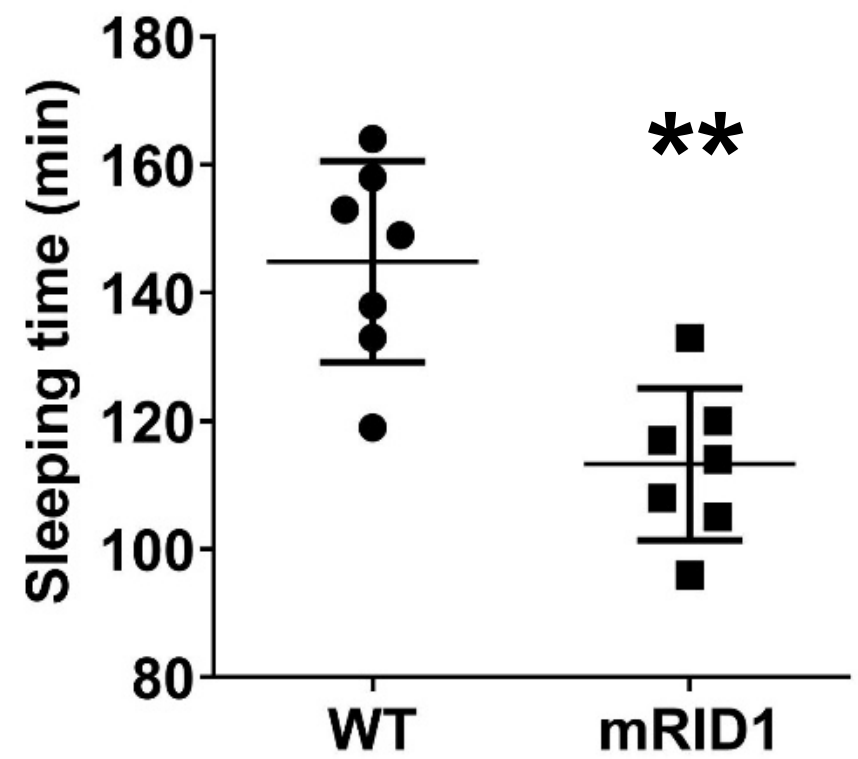

Supplementary Figure 1. HFD-fed SMRT^{mRID1} mice exhibit reduced sleep duration compared to WT mice

HFD-fed WT and SMRT^{mRID1} mice were i.p. injected with 50mg/kg phenobarbital. From the time of injection, sleep duration was recorded as the time when the mice were able to right themselves. Data are presented as mean ± STD. Statistical significance was determined by Student's *t*-test. **$p<0.01$

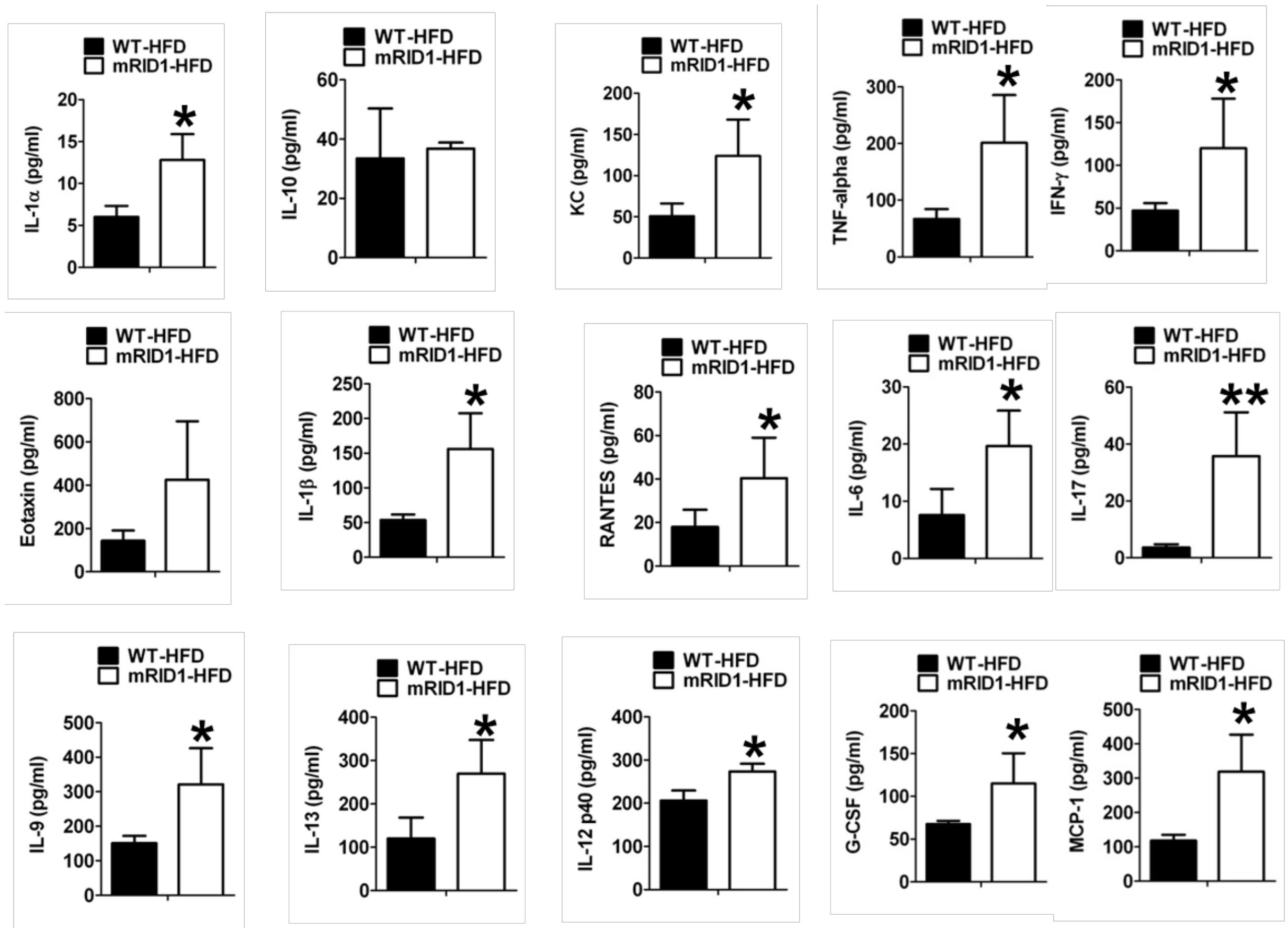

Supplementary Figure 2. Serum levels of inflammatory cytokines are elevated in HFD-fed SMRT^{mRID1} mice

Serum levels of inflammatory cytokines and chemokines were measured in HFD-fed WT and SMRT^{mRID1} mice. Concentrations of IL-1α, IL-10, KC, TNF-α, IFN-γ, eotaxin, IL-1β, RANTES, IL-6, IL-17, IL-9, IL-13, IL-12p40, G-CSF, and MCP-1 were quantified by ELISA. Data are presented as mean ± STD. Statistical significance was determined by Student's *t*-test. *$p<0.05$

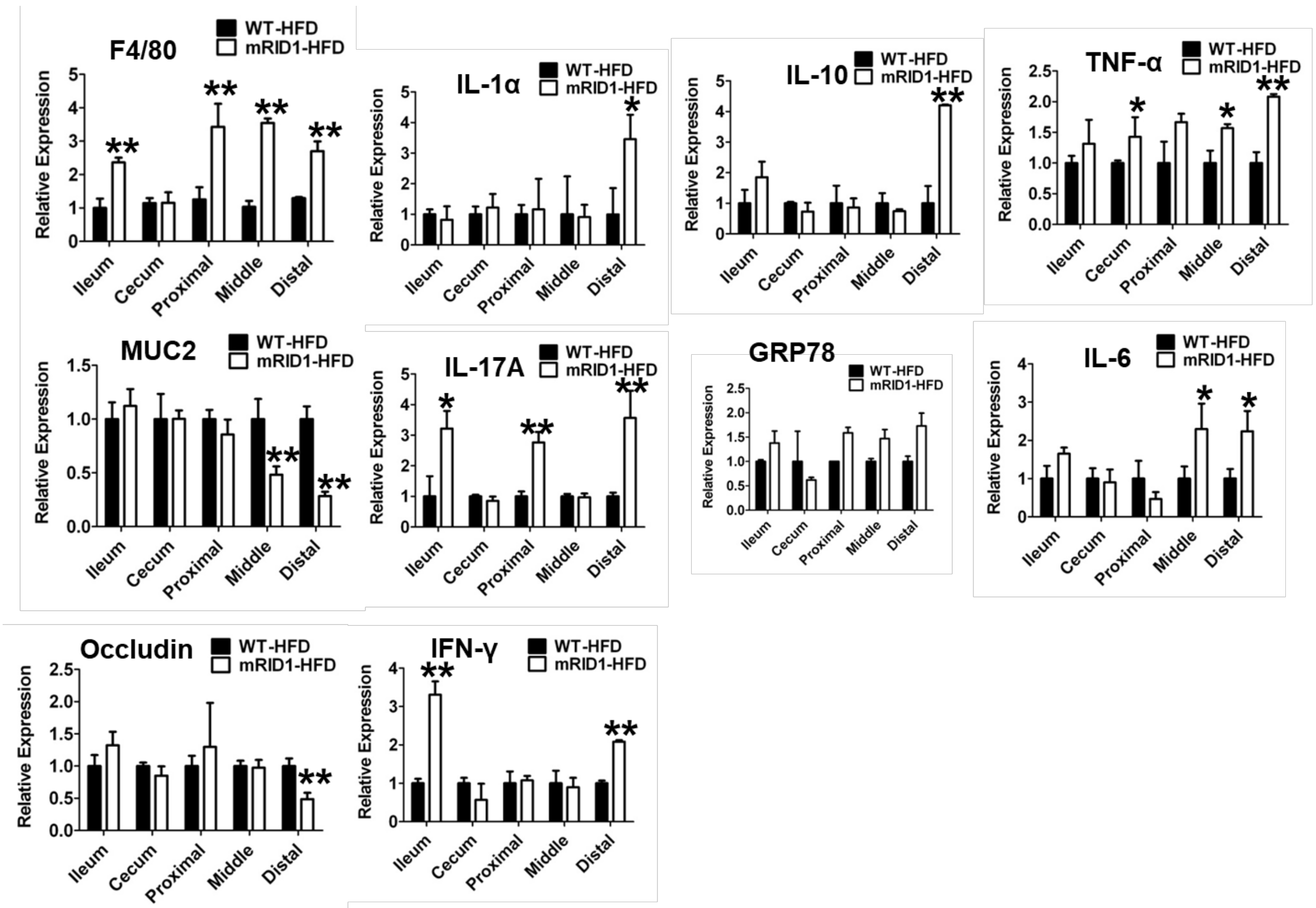

Supplementary Figure 3. Altered intestinal gene expression in HFD-fed SMRT^{mRID1} mice

Relative mRNA expression levels of macrophage marker (F4/80), inflammatory cytokines (IL-1α, IL-10, TNF-α, IL-17A, IL-6, IFN-γ), chemokine (CXCL10), and intestinal barrier–related genes (MUC2 and occludin) were measured by qPCR in different intestinal segments (ileum, cecum, proximal, middle, and distal colon) from HFD–fed WT and SMRT^{mRID1} mice. Gene expression levels were normalized to 36B4. Data are shown as mean ± STD. Statistical significance was determined by Student's t-test. *$p<0.05$, **$p<0.01$

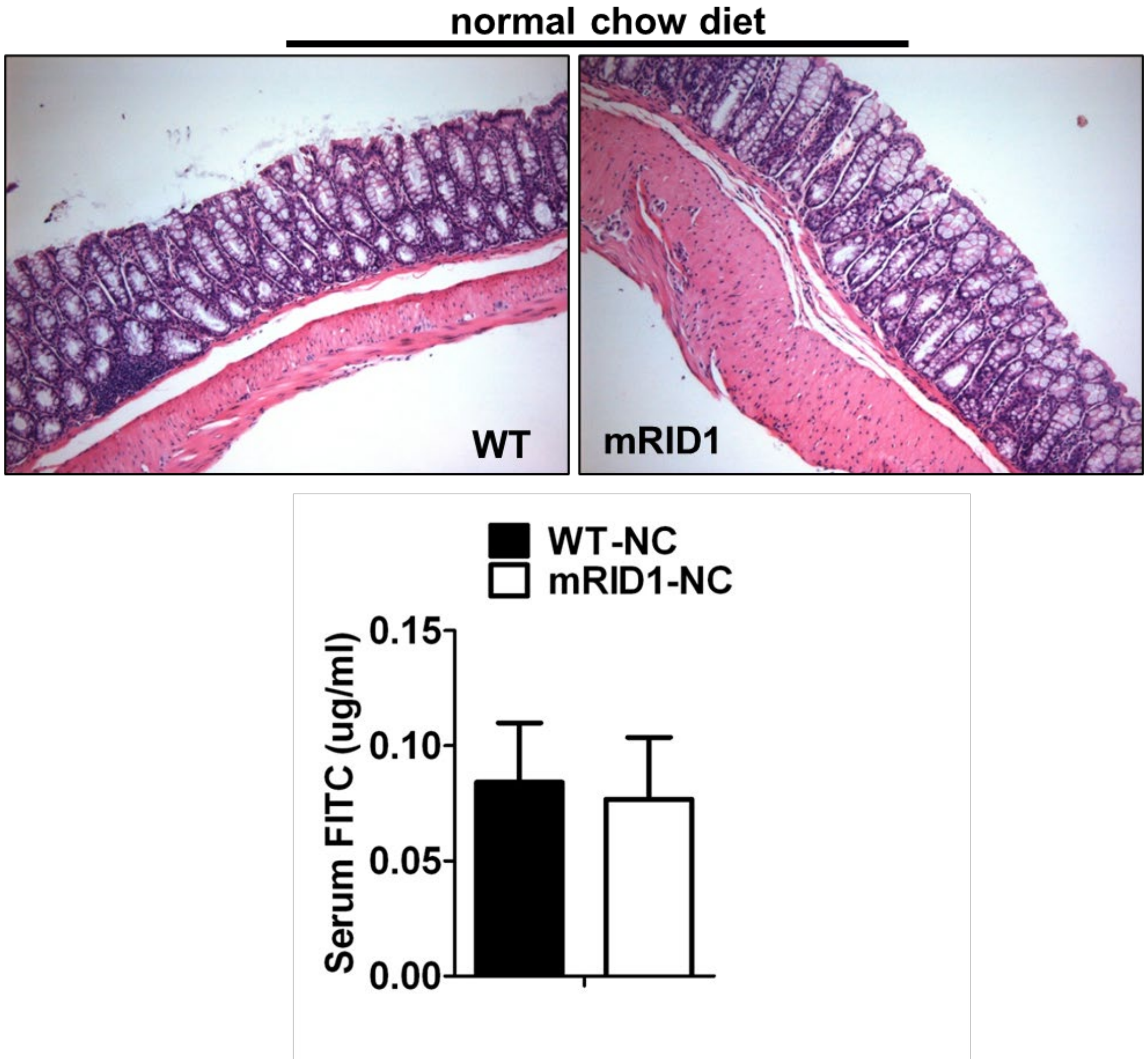

Supplementary Figure 4. Intestinal morphology and barrier function are preserved in SMRT^{mRID1} mice under normal chow conditions.

Representative H&E–stained sections of intestinal tissue from WT and SMRT^{mRID1} mice maintained on normal chow (NC) are shown (upper panels). Intestinal permeability was assessed by measuring serum FITC–dextran levels (lower panel). Data are presented as mean ± STD.

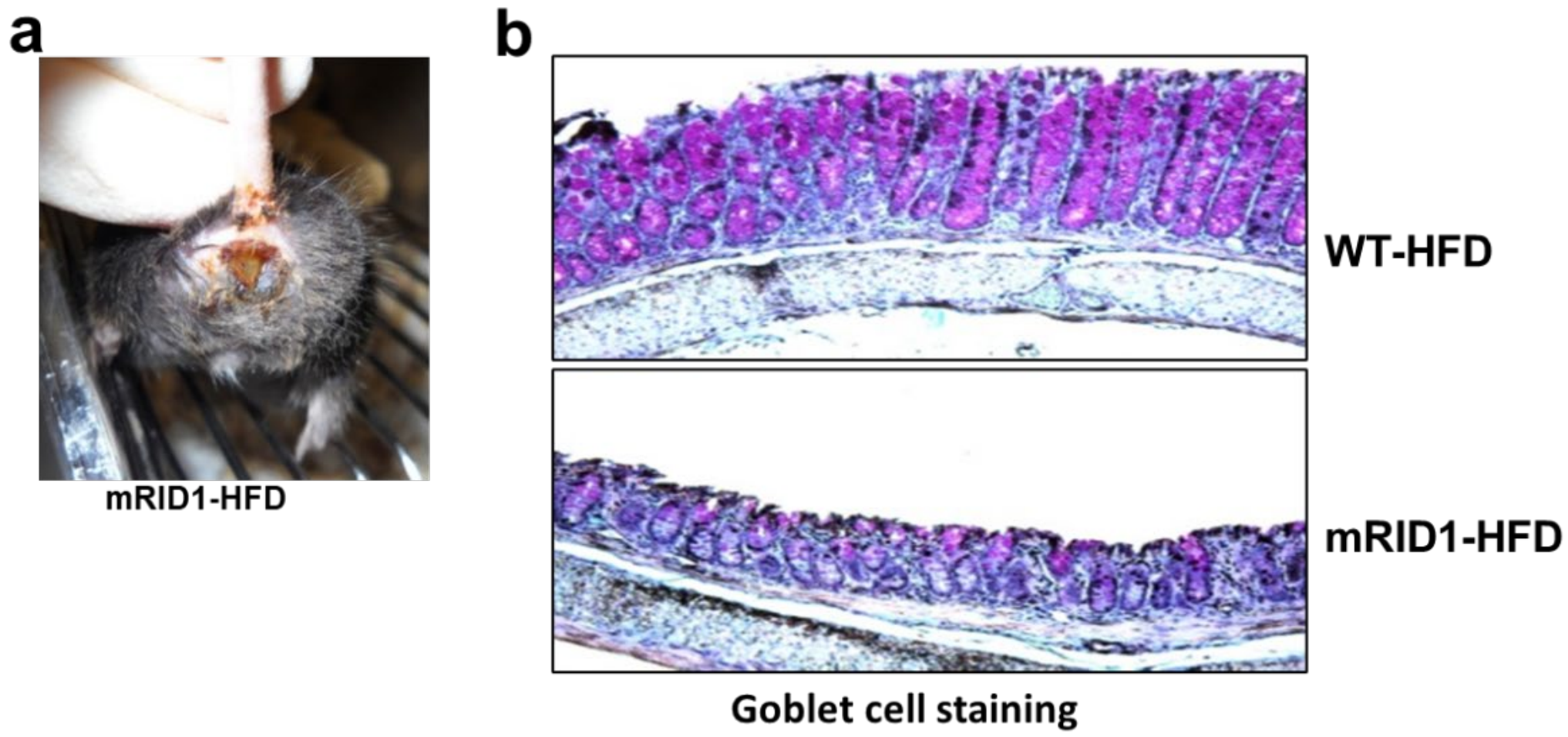

Supplementary Figure 5. Severe diarrhea and colonic epithelial damage in HFD-fed SMRT^{mRID1} mice

a, Representative image showing severe diarrhea observed in a subset of SMRT^{mRID1} mice fed a HFD. **b**, Representative histological sections of the distal colon from HFD-fed WT and SMRT^{mRID1} mice stained to visualize mucin-producing goblet cells.

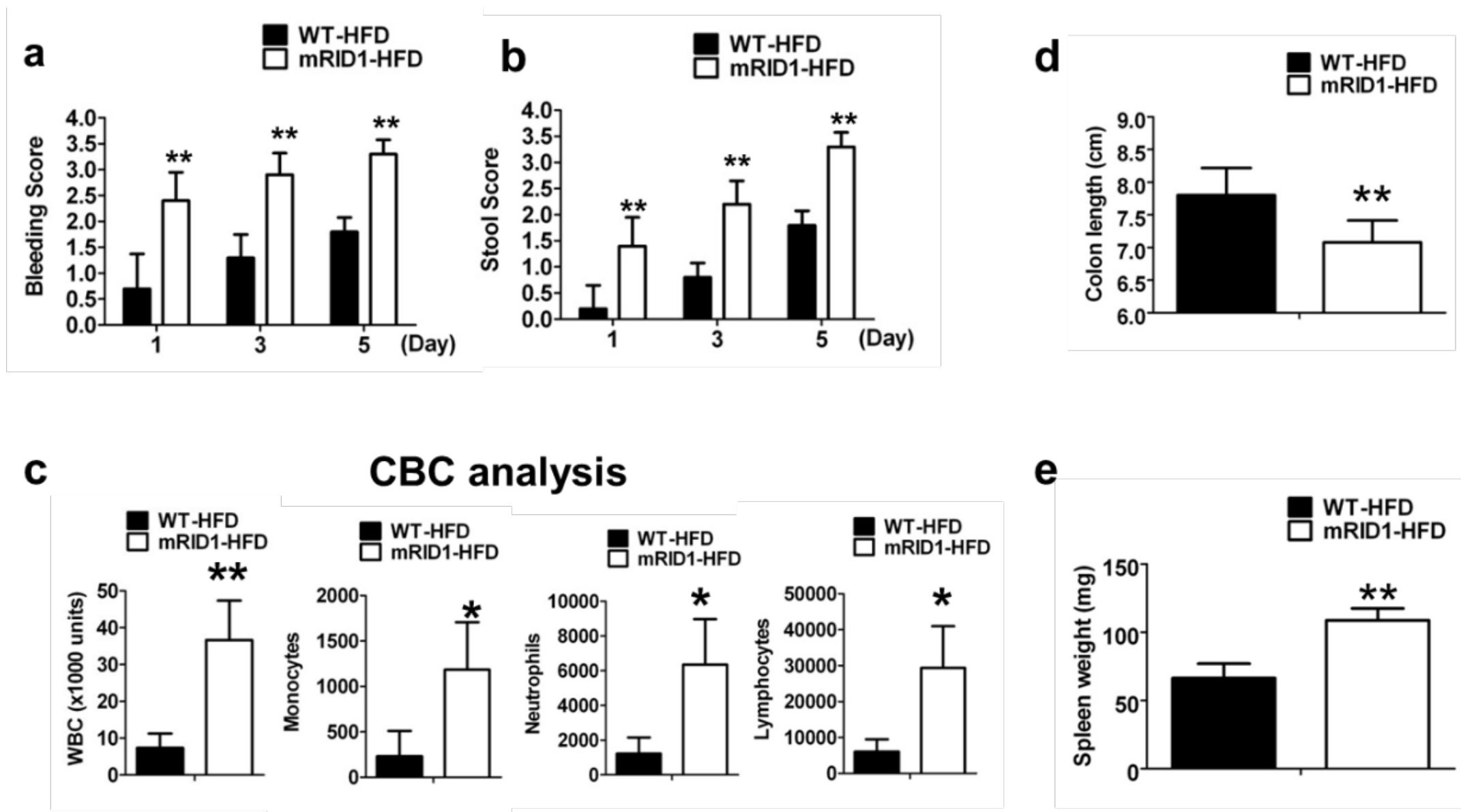

Supplementary Figure 6. HFD-fed SMRT^{mRID1} mice display increased susceptibility to DSS-induced acute colitis

HFD–fed WT and SMRT^{mRID1} mice were subjected to DSS–induced acute colitis. **a-b**, Gastrointestinal bleeding scores (**a**) and stool scores (**b**) were monitored over the course of DSS treatment. **c**, Peripheral blood leukocyte analysis revealed increased total white blood cell (WBC) counts, monocytes, neutrophils, and lymphocytes in HFD-fed WT and SMRT^{mRID1} mice. **d**, Colon length was measured at the experimental endpoint. **e**, Spleen weight of HFD-fed WT and SMRT^{mRID1} mice. Data are presented as mean ± STD. Statistical significance was determined by Student's t-test. *$p<0.05$, **$p<0.01$

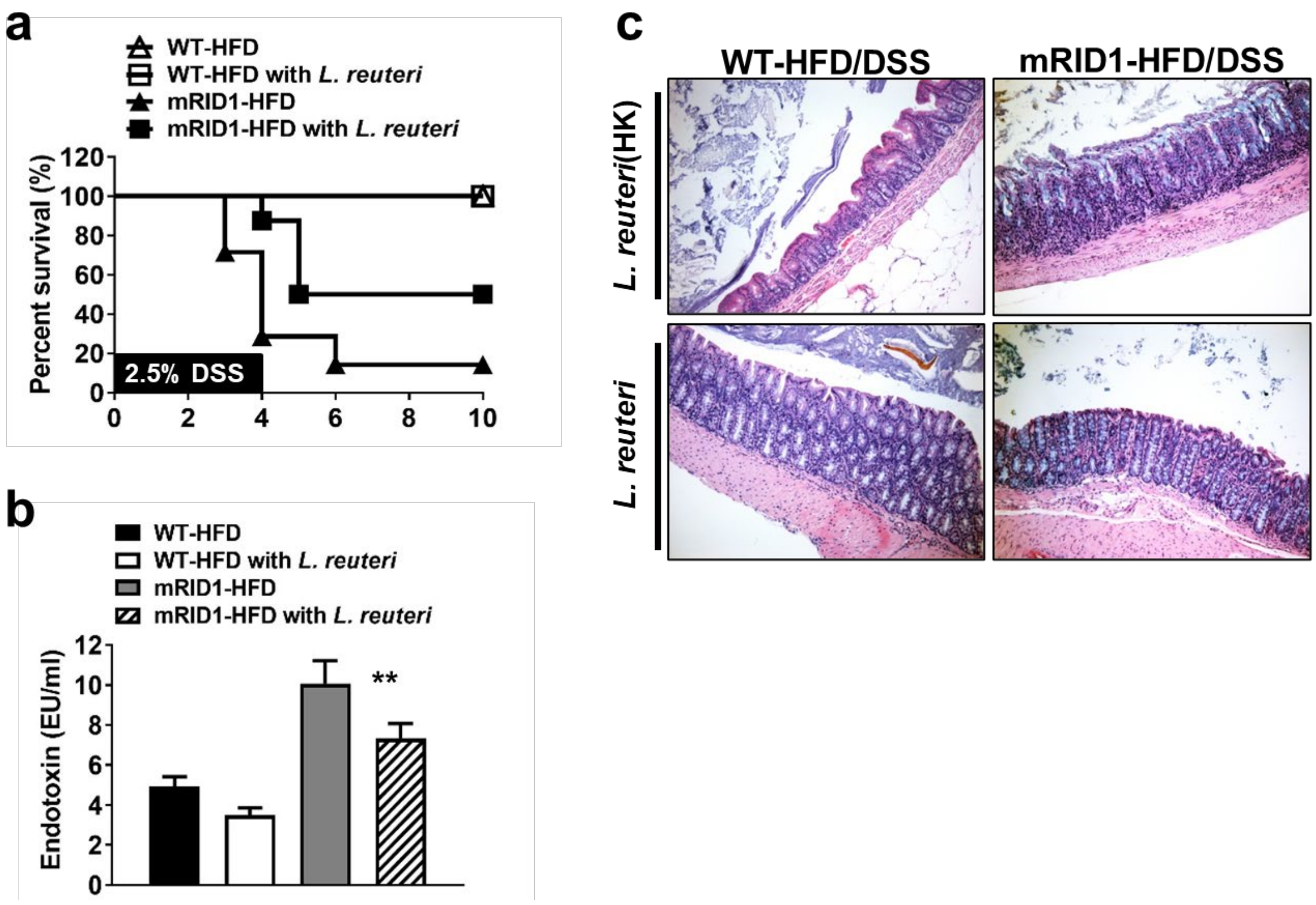

Supplementary Figure 7. *Lactobacillus reuteri* supplementation partially ameliorates DSS-induced intestinal inflammation in HFD-fed SMRT^{mRID1} mice

HFD–fed wild-type WT and SMRT^{mRID1} mice were subjected to DSS–induced acute colitis with or without *Lactobacillus reuteri* supplementation. **a**, Kaplan–Meier survival analysis during DSS treatment (2.5% DSS) with or without *L. reuteri* supplementation. **b**, Serum endotoxin levels were measured, showing a reduction in endotoxemia in *L. reuteri*–supplemented HFD-fed SMRT^{mRID1} mice. **c**, Representative histological sections of the distal colon showing reduced epithelial damage and inflammatory infiltration following *L. reuteri* supplementation. Data are presented as mean ± STD. Statistical significance was determined by one-way ANOVA.

**p<0.01

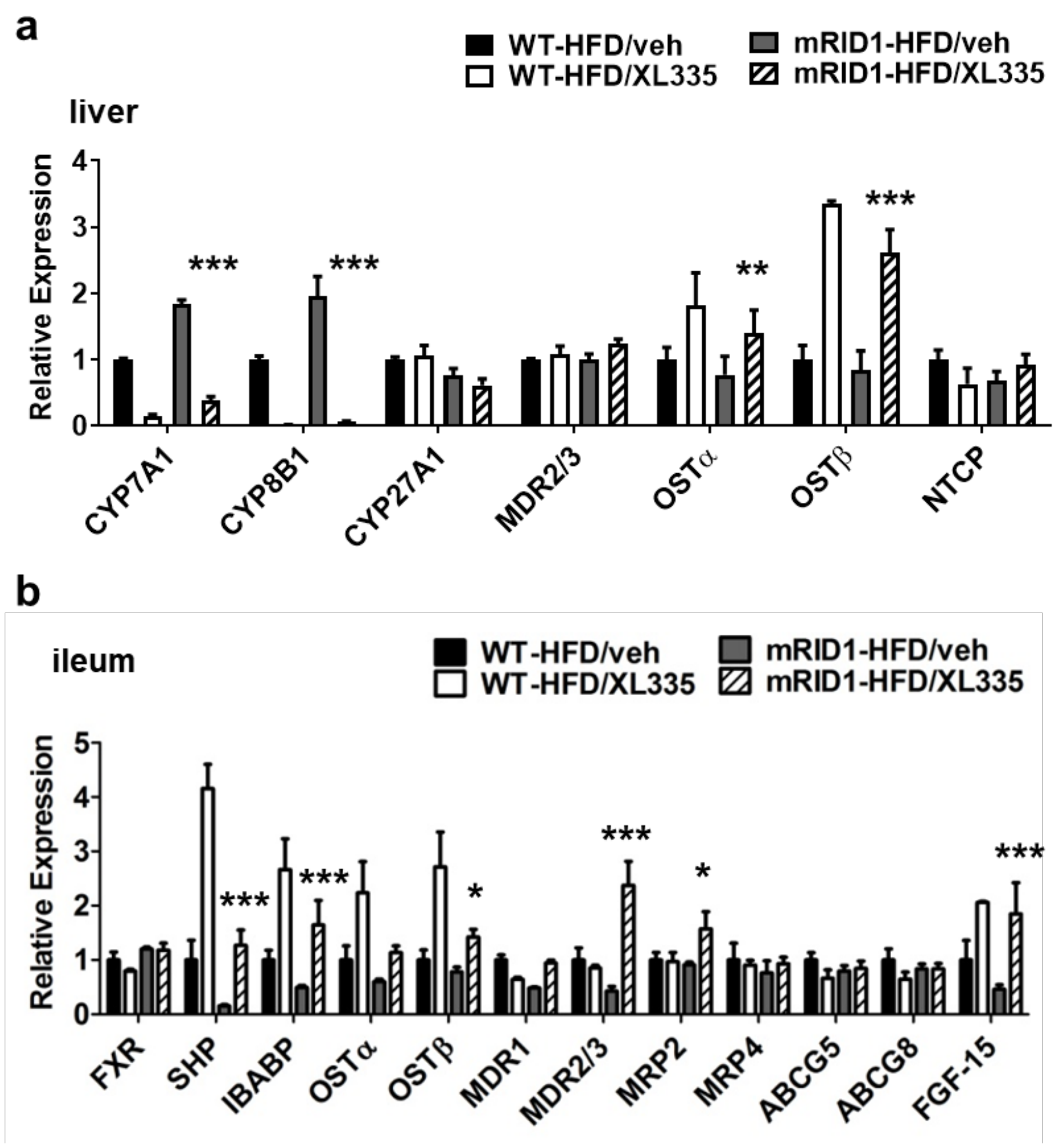

Supplementary Figure 8. FXR activation restores entero-hepatic bile acid homeostasis and mucosal defense gene expression in HFD-fed SMRT^{mRID1} mice

HFD–fed WT and SMRT^{mRID1} mice were treated daily with vehicle or the synthetic FXR agonist XL335 (30 mg/kg) for 3 weeks. **a-b**, Relative mRNA expression of genes involved in entero-hepatic BA circulation and transport was analyzed by qPCR in the liver (**a**) and ileum (**b**). Data are presented as mean ± STD. Statistical significance was determined by two-way ANOVA followed by Tukey's post hoc test. $^{*}p<0.05$, $^{**}p<0.01$, $^{***}p<0.001$

Reference

1. Xie, W., *et al.* Humanized xenobiotic response in mice expressing nuclear receptor SXR. *Nature* **406**, 435-439 (2000).
